# Wigner Time-Delay and Distribution for Polarization Interaction in Strongly Coupled Semiclassical Plasmas

**DOI:** 10.3390/e22090910

**Published:** 2020-08-19

**Authors:** Myoung-Jae Lee, Young-Dae Jung

**Affiliations:** 1 Department of Physics, Hanyang University, Seoul 04763, Korea; mjlee@hanyang.ac.kr; 2 Research Institute for Natural Sciences, Hanyang University, Seoul 04763, Korea; 3 Department of Applied Physics, Hanyang University, Ansan, Kyunggi-Do 15588, Korea

**Keywords:** Wigner time-delay, semiclassical plasma, 52.27.Gr, 52.20.Hv, 52.20.−j, 34.70.+e

## Abstract

The quantum effect on the Wigner time-delay and distribution for the polarization scattering in a semiclassical dense plasma is explored. The partial wave analysis is applied for a partially ionized dense plasma to derive the phase shift for the polarization interaction. The Wigner time-delay and the Wigner distribution are derived for the electron–atom polarization interaction including the effects of quantum-mechanical characteristic and plasma screening. In this work, we show that the Wigner time-delay and the Wigner distribution for the polarization interaction can be suppressed by the quantum effect. The Wigner time-delay and the Wigner distribution are also significantly suppressed by the increase of plasma shielding. The variation of the Wigner time-delay and the Wigner distribution function due to quantum screening is discussed.

## 1. Introduction

In atomic collisions, it has been shown that a particle scattering with the combination of the time-evolution operator and the energy dependence can provide the physical significance of the collision system in a potential field [1,2]. It is also shown that the distance shift in the reflected atomic wave packet generates the phase shift after passing the interaction region. Therefore, it is expected that the deflection of the length in the reflected wave packet will correspond to the Wigner time-delay due to the energy-time uncertainty relation since the partial wave scattering phase shift is generated by the localized potential field [3,4]. The shape of the Wigner time-delay for the resonance process takes the form of the Lorentzian function [3]. Recently, the extensive investigation has been carried out for the time-delay in photoionization processes [5,6,7]. In addition, the time-delay for the photo detachment was investigated for Yukawa potential bound electrons. In weakly coupled classical plasmas, it is well known that the Yukawa-type Debye-Hückel model is very reliable for the investigation of collision and radiation processes [8,9,10,11]. However, it is shown that the quantum-mechanical effect in a strongly coupled plasma plays an important role on the screened inter-particle interaction because of the quantum diffraction effect and the collective behavior [12,13,14,15]. It is also well-known that the polarization effect is important for the scattering of low-energy electrons by neutral atoms [16,17]. However, the quantum-mechanical effect on the Wigner time-delay for the polarization scattering in dense semiclassical plasmas has not been reported so far. Hence, we are motivated to study the influence of quantum screening on the Wigner time-delay for the polarization scattering since the it can provide the physical significance of the collective polarization interaction in a dense semiclassical plasma. In this work, the partial wave phase-shift analysis [18] is employed in order to obtain the analytic expressions of the scattering phase shift, the Wigner time-delay, and the Wigner time-delay distribution function in a partially ionized semiclassical dense plasma. The variation of the Wigner time-delay and distribution function due to the quantum and plasma shielding effects is also discussed.

## 2. Theory and Calculations

In a weakly coupled plasma, the effective attractive polarization interaction $V_{B}(r,r_{D})$ between the projectile electron and the neutral atom is obtained as the Buckingham-type form [17].

(1) $$V_{B}(r,r_{D})=-\frac{e^{2}\alpha }{2r^{4}}{\left(1+\frac{r}{r_{D}}\right)}^{2}\exp\left(-\frac{2r}{r_{D}}\right),$$

where α is the dipole polarizability of the neutral atom and $r_{D}[={\left(k_{B}T_{e}/4\pi n_{e}e^{2}\right)}^{1/2}]$ is the standard Debye length with $k_{B}$, $T_{e}$, and $n_{e}$ being Boltzmann’s constant, the electron temperature, and the electron density, respectively. The effective pseudopotential $V_{\mathrm{RDO}}(r,\lambda ,r_{D})$ for the polarization electron-atom interaction encompassing the quantum-mechanical effect was derived by Ramazanov-Dzhumagulova-Omarbakiyeva (RDO) [13] in a strongly-coupled semiclassical plasma.

(2) $$\begin{array}{ll} V_{\mathrm{RDO}}(r,\lambda ,r_{D})= & -\frac{e^{2}\alpha }{2r^{4}{\left(1-4\lambda^{2}/r_{D}^{2}\right)}^{1/2}}\{\left[rB\left(\lambda ,r_{D}\right)+1\right]e^{-rB(\lambda ,r_{D})}\\ & -{\left[rA(\lambda ,r_{D})+1\right]e^{-rA(\lambda ,r_{D})}\}}^{2} \end{array}$$

where $\lambda [=\hbar {\left(\pi m_{e}k_{B}T\right)}^{-1/2}]$ is the electron de Broglie wavelength, ℏ is the Planck constant divided by 2π, and $m_{e}$ is the electron mass. In Equation (2), the quantum screening (*B*, *A*) parameters [13] are represented as follows: $B(\lambda ,r_{D})\equiv {\left[1-{\left(1-4\lambda^{2}/r_{D}^{2}\right)}^{1/2}\right]}^{1/2}/{\left(2\lambda^{2}\right)}^{1/2}$ and $A(\lambda ,r_{D})\equiv {\left[1+{\left(1-4\lambda^{2}/r_{D}^{2}\right)}^{1/2}\right]}^{1/2}/{\left(2\lambda^{2}\right)}^{1/2}$. Then, the constraint of the range of the electron de Broglie wavelength $2\lambda <r_{D}$ can be obtain by the quantum screening parameters $B(\lambda ,r_{D}){\left(2\lambda^{2}\right)}^{1/2}={\left[1-{\left(1-4\lambda^{2}/r_{D}^{2}\right)}^{1/2}\right]}^{1/2}$ and $A(\lambda ,r_{D}){\left(2\lambda^{2}\right)}^{1/2}={\left[1+{\left(1-4\lambda^{2}/r_{D}^{2}\right)}^{1/2}\right]}^{1/2}$. Hence, we retain this condition $2\lambda <r_{D}$ throughout in this work. When there is no quantum-mechanical effect in a plasma, the pseudopotential $V_{\mathrm{RDO}}(r,\lambda ,r_{D})$ becomes the Buckingham potential $V_{B}(r,r_{D})$ (Equation (2)), i.e., $V_{\mathrm{RDO}}(r,\lambda \to 0,r_{D})\to V_{B}(r,r_{D})=-(e^{2}\alpha /2r^{4}){\left(1+r/r_{D}\right)}^{2}\exp(-2r/r_{D})$, since the quantum screening (*B*, *A*) parameters are $B(\lambda \to 0,r_{D})\to 1/r_{D}$ and $A(\lambda \to 0,r_{D})\to \infty$. It is well-known that the Buckingham potential has been widely used for the description of the electron-atom collisions by the polarization interaction [16]. The analytic expression of the effective pseudopotential obtained by Ramazanov, Dzhumagulova, and Omarbakiyeva [13] has the Buckingham-type form including the quantum-mechanical effect. Hence, it would be expected that the investigation using the Ramazanov-Dzhumagulova-Omarbakiyeva (RDO) potential $V_{\mathrm{RDO}}(r,\lambda ,r_{D})$ (Equation (2)) can provide the precise expression of the influence of quantum screening on the atomic collision cross section and the excitation rate in a semiclassical dense plasma since the difference between the standard Buckingham potential and Ramazanov-Dzhumagulova-Omarbakiyeva potential generates the pure quantum effects in a semiclassical dense plasma. The radial Schrödinger equation for a given potential V(r) is given by the equation below.

(3) $$\left[\frac{1}{r^{2}}\frac{d}{dr}\left(r^{2}\frac{d}{dr}\right)-\frac{\ell \left(\ell +1\right)}{r^{2}}-\frac{2\mu }{\hbar^{2}}V\left(r\right)+k^{2}\right]R_{\ell }(k;r)=0,$$

where ℓ is the angular-momentum quantum number, $k[={\left(2m_{e}E/\hbar^{2}\right)}^{1/2}]$ is the wave number with $E(=m_{e}v^{2}/2)$, $v(=\hbar k/m_{e})$ being the scattering energy and the group velocity of the wave packet, respectively. In this case, the solution $R_{\ell }(k,r)$ is found to be the following [19,20].

(4) $$\begin{array}{ll} R_{l}(k,r)=j_{l}(kr)+\frac{2m_{e}k}{\hbar^{2}} & [\int_{\begin{array}{l} r \end{array}}^{\begin{array}{l} \infty \end{array}}dr^{\prime }{r^{\prime }}^{2}R_{l}(k,r^{\prime })n_{l}(kr^{\prime })V(r^{\prime })j_{l}(kr)\\ & +\int_{\begin{array}{l} 0 \end{array}}^{\begin{array}{l} r \end{array}}dr^{\prime }{r^{\prime }}^{2}R_{l}(k,r^{\prime })j_{l}(kr^{\prime })V(r^{\prime })n_{l}(kr)], \end{array}$$

where $j_{l}(kr)$ and $n_{l}(kr)$ are the spherical Bessel and the Neumann functions. Hence, the asymptotic expression [19] of the radial solution can be written as $R_{l}(k,r)\propto {\left(kr\right)}^{-1}\sin[kr+\delta_{l}(k)-l\pi /2]$, where $\delta_{l}(k)$ is the partial phase-shift. Based on the phase-shift analysis, the *l*th-order partial wave scattering phase shift [20,21] $\delta_{l}(k)$ for the low-energy scattering in a potential field V(r) can be written by the equation below.

(5) $$\delta_{\ell }(k)\approx -\frac{\pi m_{e}}{\hbar^{2}}\int_{\begin{array}{l} r_{L} \end{array}}^{\begin{array}{l} r_{U} \end{array}}dr\;rJ_{\ell +1/2}^{2}(kr)V(r),$$

where $r_{U}$ and $r_{L}$ are, respectively, the upper-cutoff for the interaction domain and the lower-cutoff for the distance of the closest encounter and $J_{q}(kr)$ is the first type of Bessel function. From Equations (2) and (5), the zeroth-order phase shift $\delta_{\mathrm{RDO}}$ for the Ramazanov-Dzhumagulova-Omarbakiyeva (RDO) screened polarization electron-atom interaction is obtained by the equation below.

(6) $$\begin{array}{ll} \delta_{\mathrm{RDO}}(k,\lambda ,r_{D})≅ & -\frac{\alpha }{3a_{0}}\{\pi k^{2}-2B^{2}(\lambda ,r_{D}){\left[k^{2}/B^{2}(\lambda ,r_{D})-1\right]}^{1/2}\\ & -2k^{2}\tan^{-1}{\left[k^{2}/B^{2}(\lambda ,r_{D})-1\right]}^{-1/2}-4B^{2}(\lambda ,r_{D})\sec^{-1}\left(k/B(\lambda ,r_{D})\right)\}, \end{array}$$

where $a_{0}(=\hbar^{2}/m_{e}e^{2})$ is the first Bohr radius since $r_{U}={\left(2B\right)}^{-1}$, $r_{L}={\left(2k\right)}^{-1}$, $V_{RDO}(r,\lambda \to 0,r_{D})\to V_{B}(r,r_{D})$, and $B(\lambda ,r_{D})<<A(\lambda ,r_{D})$ for $2\lambda <r_{D}$. Since the upper-cutoff $r_{U}$ for the interaction domain and the lower-cutoff $r_{L}$ for the distance of the closest are, respectively, given by $r_{U}={\left(2\lambda^{2}\right)}^{1/2}/2{\left[1-{\left(1-4\lambda^{2}/r_{D}^{2}\right)}^{1/2}\right]}^{1/2}$ and $r_{L}=1/2k$, the upper-cutoff for the interaction range is very sensitive to the influence of quantum screening.

In a weakly coupled classical plasma described by the standard Yukawa-exponential term $\exp(-r/r_{D})$, the infarction range has been usually defined as $r=r_{D}$. Hence, the main Ramazanov-Dzhumagulova-Omarbakiyeva-exponential term in Equation (2) is $\exp[-rB(\lambda ,r_{D})]$ since $B(\lambda ,r_{D})<<A(\lambda ,r_{D})$. Hence, the choice of the upper-cutoff in Equation (5), $r_{U}(\lambda ,r_{D})=1/B(\lambda ,r_{D})={\left(2\lambda^{2}\right)}^{1/2}/2{\left[1-{\left(1-4\lambda^{2}/r_{D}^{2}\right)}^{1/2}\right]}^{1/2}$, is very reliable since the upper-cutoff becomes $r_{U}(\lambda \to 0,r_{D})=r_{D}$ in λ→0, which is identical to the case of a weakly coupled classical plasma. When there is no influence of quantum diffraction and plasma screening such as λ→0 and $r_{D}\to \infty$, the zeroth-order phase shift [22] $\delta_{0}(k)$ is then given by $\delta_{0}(k)=-\pi \alpha k^{2}/3a_{0}$, which is the case of the free polarization interaction $V_{pol}(r)=-e^{2}\alpha /2r^{4}$. The Wigner time-delay [3,4,23], $t_{\mathrm{Wigner}}$ can be represented by the energy (*E*) derivative of the phase shift such as:

(7) $$t_{\mathrm{Wigner}}=\hbar \frac{d\delta }{dE}=\frac{m_{e}}{\hbar k}\frac{d\delta }{dk},$$

since $E=\hbar^{2}k^{2}/2m_{e}$ and t↔iℏ∂/∂E is due to the energy-time uncertainty relation. From Equations (6) and (7), the scaled Wigner time-delay ${\bar{t}}_{\mathrm{Wigner}}(=t_{\mathrm{Wigner}}/t_{0})$ in units of $t_{0}\left(\equiv -\pi \alpha /3a_{0}\right)$ for the polarization electron-atom interaction in partially ionized dense semiclassical plasmas is then found to be:

(8) $$\begin{array}{ll} {\bar{t}}_{\mathrm{Wigner}}(\bar{E},\bar{\lambda },{\bar{r}}_{D})= & 1-{\left[2\bar{E}/{\bar{B}}^{2}(\bar{\lambda },{\bar{r}}_{D})\right]}^{-1}{\left[\bar{E}/{\bar{B}}^{2}(\bar{\lambda },{\bar{r}}_{D})-1\right]}^{-1/2}\\ & -\frac{2}{\pi }\tan^{-1}{\left[\bar{E}/{\bar{B}}^{2}(\bar{\lambda },{\bar{r}}_{D})-1\right]}^{-1/2}\\ & =1-{\left[2\bar{E}{\bar{\lambda }}^{2}{\left[1-{\left(1-4{\bar{\lambda }}^{2}/{\bar{r}}_{D}^{2}\right)}^{1/2}\right]}^{-1}\right]}^{-1}\\ & \times {\left[2\bar{E}{\bar{\lambda }}^{2}{\left[1-{\left(1-4{\bar{\lambda }}^{2}/{\bar{r}}_{D}^{2}\right)}^{1/2}\right]}^{-1}-1\right]}^{-1/2}\\ & -\frac{2}{\pi }\tan^{-1}{\left[2\bar{E}{\bar{\lambda }}^{2}{\left[1-{\left(1-4{\bar{\lambda }}^{2}/{\bar{r}}_{D}^{2}\right)}^{1/2}\right]}^{-1}-1\right]}^{-1/2}, \end{array}$$

where $\bar{E}(\equiv E/Ry)={\bar{k}}^{2}$ is the scattering energy scaled by the Rydberg constant $Ry(=m_{e}e^{4}/2\hbar^{2}\approx 13.6\;\mathrm{eV})$, $\bar{k}(\equiv k/a_{0})$ is the scaled wave number, $\bar{\lambda }(\equiv \lambda /a_{0})$ is the scaled electron de Broglie wavelength, ${\bar{r}}_{D}(\equiv r_{D}/a_{0})$ is the scaled Debye radius, and $\bar{B}(\bar{\lambda },{\bar{r}}_{D})[\equiv B(\lambda ,r_{D})/a_{0}]={\left[1-{\left(1-4{\bar{\lambda }}^{2}/{\bar{r}}_{D}^{2}\right)}^{1/2}\right]}^{1/2}/{\left(2{\bar{\lambda }}^{2}\right)}^{1/2}$. It is shown that the time delay representation for the Brownian type motion allows the exponential function of the distribution [24,25]. Hence, the Wigner distribution for the time-delay $P({\bar{t}}_{\mathrm{Wigner}})$ is represented as follows:

(9) $$P(\bar{k},{\bar{t}}_{\mathrm{Wigner}})=\frac{{\bar{r}}_{D}}{2\bar{k}{\bar{t}}_{\mathrm{Wigner}}^{2}}\exp\left(-\frac{{\bar{r}}_{D}}{2\bar{k}{\bar{t}}_{\mathrm{Wigner}}^{2}}\right),$$

since the localized screening domain can be substituted by the Debye length of the plasma system. Without quantum and plasma screening effects, i.e., λ→0 and $r_{D}\to \infty$, the Wigner time-delay distribution $P_{0}\equiv P(\bar{k},\bar{\lambda }\to 0,{\bar{r}}_{D}\to \infty )$ would be $P(\bar{k},\bar{\lambda }\to 0,{\bar{r}}_{D}\to \infty )=({\bar{r}}_{D}/2\bar{k})\exp(-{\bar{r}}_{D}/2\bar{k})$. Hence, the scaled Wigner time-delay distribution ${\bar{P}}_{\mathrm{Wigner}}(\bar{E},\bar{\lambda },{\bar{r}}_{D})\equiv P({\bar{t}}_{\mathrm{Wigner}})/P_{0}$ in units of $P_{0}$ for the polarization electron-atom scattering in partially ionized dense semiclassical plasmas becomes the following.

(10) $$\begin{array}{ll} {\bar{P}}_{\mathrm{Wigner}}(\bar{E},\bar{\lambda },{\bar{r}}_{D})= & \frac{1}{{\bar{t}}_{\mathrm{Wigner}}^{2}(\bar{E},\bar{\lambda },{\bar{r}}_{D})}\exp\left[-\frac{{\bar{r}}_{D}}{2{\bar{E}}^{1/2}}\left(\frac{1}{{\bar{t}}_{\mathrm{Wigner}}^{2}(\bar{E},\bar{\lambda },{\bar{r}}_{D})}-1\right)\right]\\ & =\{1-{\left[2\bar{E}{\bar{\lambda }}^{2}{\left[1-{\left(1-4{\bar{\lambda }}^{2}/{\bar{r}}_{D}^{2}\right)}^{1/2}\right]}^{-1}\right]}^{-1}\\ & \times {\left[2\bar{E}{\bar{\lambda }}^{2}{\left[1-{\left(1-4{\bar{\lambda }}^{2}/{\bar{r}}_{D}^{2}\right)}^{1/2}\right]}^{-1}-1\right]}^{-1/2}\\ & {-\frac{2}{\pi }\tan^{-1}{\left[2\bar{E}{\bar{\lambda }}^{2}{\left[1-{\left(1-4{\bar{\lambda }}^{2}/{\bar{r}}_{D}^{2}\right)}^{1/2}\right]}^{-1}-1\right]}^{-1/2}\}}^{-2}\\ & \times \exp\{-\frac{{\bar{r}}_{D}}{2{\bar{E}}^{1/2}}[(1-{\left[2\bar{E}{\bar{\lambda }}^{2}{\left[1-{\left(1-4{\bar{\lambda }}^{2}/{\bar{r}}_{D}^{2}\right)}^{1/2}\right]}^{-1}\right]}^{-1}\\ & \times {\left[2\bar{E}{\bar{\lambda }}^{2}{\left[1-{\left(1-4{\bar{\lambda }}^{2}/{\bar{r}}_{D}^{2}\right)}^{1/2}\right]}^{-1}-1\right]}^{-1/2}\\ & {-\frac{2}{\pi }\tan^{-1}{\left[2\bar{E}{\bar{\lambda }}^{2}{\left[1-{\left(1-4{\bar{\lambda }}^{2}/{\bar{r}}_{D}^{2}\right)}^{1/2}\right]}^{-1}-1\right]}^{-1/2})}^{-2}-1]\}. \end{array}$$

As seen in Equation (10), the deviation from the unity $\Delta [\equiv 1/{\bar{t}}_{\mathrm{Wigner}}^{2}(\bar{E},\bar{\lambda },{\bar{r}}_{D})-1]$ in the exponent represents the influence of a quantum shielding effect on the Wigner time-delay distribution tail. The analytic expressions for the Wigner time-delay [Equation (8)] and the Wigner time-delay distribution (Equation (10)) are our main results since the influence of quantum shielding on the Wigner time-delay and the Wigner time-delay distribution can be readily obtained by ${\bar{t}}_{\mathrm{Wigner}}(\bar{E},\bar{\lambda },{\bar{r}}_{D})/{\bar{t}}_{\mathrm{Wigner}}(\bar{E},\bar{\lambda }\to 0,{\bar{r}}_{D})$ and ${\bar{P}}_{\mathrm{Wigner}}(\bar{E},\bar{\lambda },{\bar{r}}_{D})/{\bar{P}}_{\mathrm{Wigner}}(\bar{E},\bar{\lambda }\to 0,{\bar{r}}_{D})$, respectively. Then, it would be clear that our analytic results for the Wigner time-delay (Equation (8)) and the Wigner time-delay distribution (Equation (10)) are more convenient than the numerical results since our analytic results can be directly used in the collisional-radiative plasma spectroscopic codes in dense plasmas. Hence, we retain the analytic investigation throughout this work. Recently, the degenerate quantum plasma has been extensively studied including the effects of Bohm pressure, electron exchange-correlation, and quantum recoil [26,27,28,29,30,31]. Therefore, those effects on the Wigner time-delay for the scattering process in a degenerate quantum plasma will be investigated elsewhere.

## 3. Discussions

**Figure 1:**
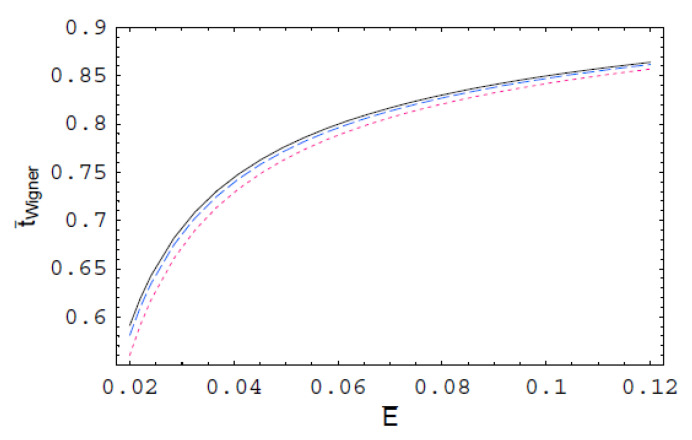
The scaled Wigner time-delay ${\bar{t}}_{\mathrm{Wigner}}$ for the polarization interaction in semiclassical plasmas as a function of the scaled collision energy $\bar{E}$ for ${\bar{r}}_{D}=10$. The solid line represents the scaled Wigner time-delay for $\bar{\lambda }=1$. The dashed line represents the scaled Wigner time-delay for $\bar{\lambda }=2$. The dotted line represents the scaled Wigner time-delay for $\bar{\lambda }=3$.

**Figure 2:**
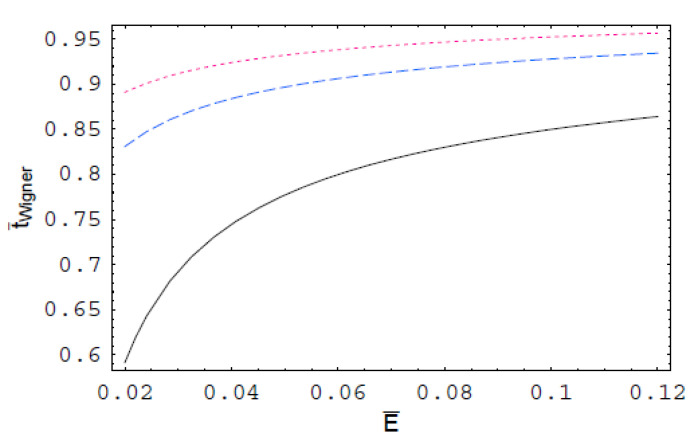
The scaled Wigner time-delay ${\bar{t}}_{\mathrm{Wigner}}$ for the polarization interaction in semiclassical plasmas as a function of the scaled collision energy $\bar{E}$ for $\bar{\lambda }=1$. The solid line represents the scaled Wigner time-delay for ${\bar{r}}_{D}=10$. The dashed line represents the scaled Wigner time-delay for ${\bar{r}}_{D}=20$. The dotted line represents the scaled Wigner time-delay for ${\bar{r}}_{D}=30$.

**Figure 3:**
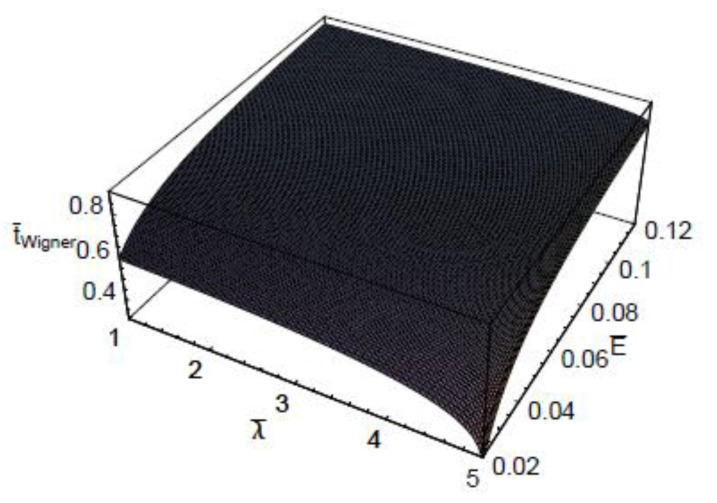
The three-dimensional plot of the scaled Wigner time-delay ${\bar{t}}_{\mathrm{Wigner}}$ as a function of the scaled de Broglie wavelength $\bar{\lambda }$ and the scaled collision energy $\bar{E}$ for ${\bar{r}}_{D}=10$.

**Figure 4:**
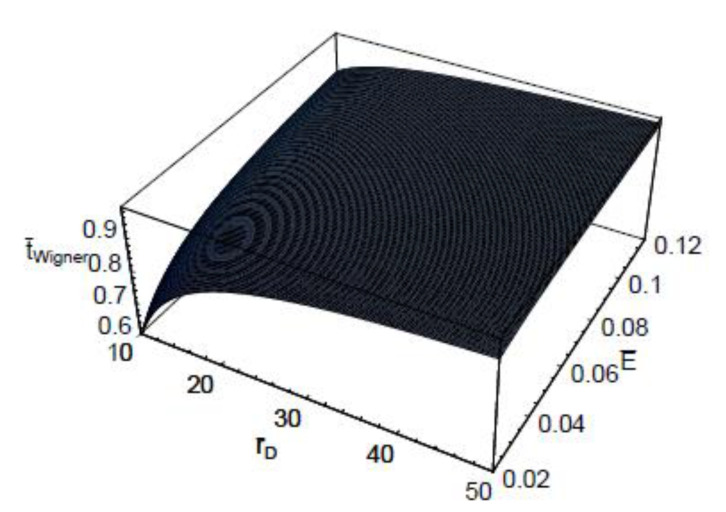
The three-dimensional plot of the scaled Wigner time-delay ${\bar{t}}_{\mathrm{Wigner}}$ as a function of the scaled Debye length ${\bar{r}}_{D}$ and the scaled collision energy $\bar{E}$ for $\bar{\lambda }=1$.

**Figure 5:**
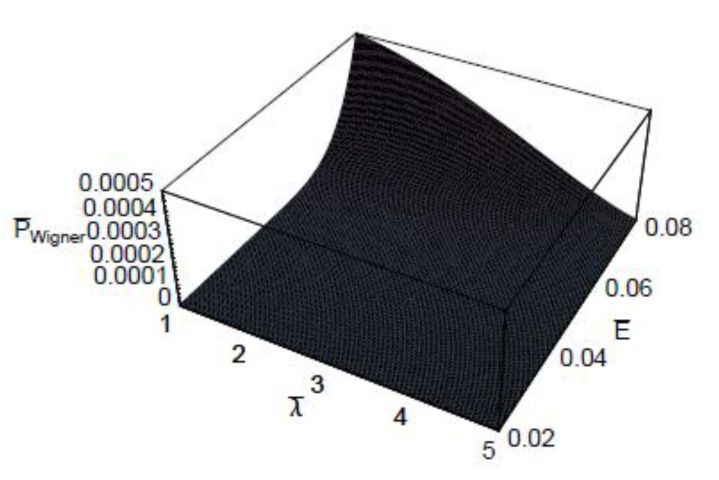
The three-dimensional plot of the scaled Wigner distribution function ${\bar{P}}_{\mathrm{Wigner}}$ as a function of the scaled de Broglie wavelength $\bar{\lambda }$ and the scaled collision energy $\bar{E}$ for ${\bar{r}}_{D}=10$.

**Figure 6:**
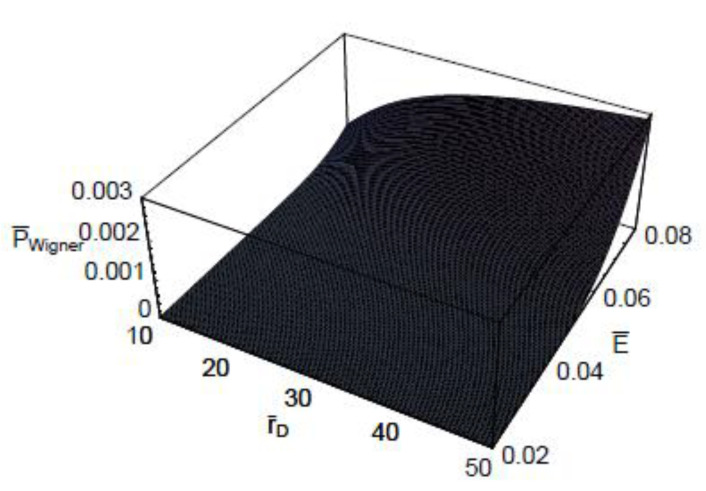
The three-dimensional plot of the scaled Wigner distribution function ${\bar{P}}_{\mathrm{Wigner}}$ as a function of the scaled Debye length ${\bar{r}}_{D}$ and the scaled collision energy $\bar{E}$ for $\bar{\lambda }=1$.

Figure 1 indicates the scaled Wigner time-delay ${\bar{t}}_{\mathrm{Wigner}}$ for the polarization interaction in a dense semiclassical plasma as a function of the scaled scattering energy $\bar{E}$ for various values of the scaled de Broglie wavelength $\bar{\lambda }$. As we can see, ${\bar{t}}_{\mathrm{Wigner}}$ increases with an increase of $\bar{E}$. It is also shown that ${\bar{t}}_{\mathrm{Wigner}}$ decreases with an increase of $\bar{\lambda }$. Thus, we found that the Wigner time-delay for the polarization interaction in a strongly coupled plasma is suppressed by the quantum-mechanical effect. In Figure 2, ${\bar{t}}_{\mathrm{Wigner}}$ is plotted as a function of $\bar{E}$ for various values of the scaled Debye length ${\bar{r}}_{D}$, which shows that it is strongly reduced by the plasma shielding effect. It is also shown that the plasma screening effect on ${\bar{t}}_{\mathrm{Wigner}}$ decreases with an increase of $\bar{E}$ Figure 3 shows the three-dimensional plot of ${\bar{t}}_{\mathrm{Wigner}}$ as a function of $\bar{\lambda }$ and $\bar{E}$. As shown in this figure, the dependence of the quantum-mechanical effect on the scaled Wigner time-delay is more significant for small collision energies. It is also shown that the energy dependence on the scaled Wigner time-delay ${\bar{t}}_{\mathrm{Wigner}}$ is more sensitive in the large de Broglie wavelength domains. Figure 4 represents the three-dimensional plot of the scaled Wigner time-delay ${\bar{t}}_{\mathrm{Wigner}}$ as a function of the scaled Debye length ${\bar{r}}_{D}$ and the scaled collision energy $\bar{E}$. As seen, the dependence of the plasma shielding effect on the scaled Wigner time-delay ${\bar{t}}_{\mathrm{Wigner}}$ is found to be more significant for small collision energies. It is also shown that the energy dependence on the scaled Wigner time-delay is more sensitive in the small Debye length domains. Figure 5 depicts the three-dimensional plot of the scaled Wigner distribution function ${\bar{P}}_{\mathrm{Wigner}}$ as a function of $\bar{\lambda }$ and $\bar{E}$. As we can see in this figure, ${\bar{P}}_{\mathrm{Wigner}}$ decreases with an increase of $\bar{\lambda }$, which indicates that the Wigner distribution is reduced by the quantum-mechanical effect. It is also shown that ${\bar{P}}_{\mathrm{Wigner}}$ increases with an increase of $\bar{E}$. Figure 6 represents the three-dimensional plot of ${\bar{P}}_{\mathrm{Wigner}}$ as a function of ${\bar{r}}_{D}$ and $\bar{E}$. As shown in this figure, the value of the Wigner distribution function increases with a growth of the Debye length. This implies that the Wigner distribution can be suppressed by the plasma screening effect. It is shown that the characteristic properties of dense plasma [13] would be represented by the density parameter $r_{s}(=a/a_{0})$, degeneracy parameter $\theta (=k_{B}T/E_{F})$, and electron plasma coupling parameter $\Gamma (=e^{2}/ak_{B}T)$, where *a* is the average distance between plasma particles, and $E_{F}$ is the Fermi energy. Hence, the plasma coupling parameter is proportional to the square of the electron de Broglie wavelength. Therefore, it would be expected that the scaled Wigner time-delay ${\bar{t}}_{\mathrm{Wigner}}$ decreases with an increase of the plasma coupling parameter Γ. Moreover, it would be also expected that ${\bar{P}}_{\mathrm{Wigner}}$ decreases with an increase of the plasma coupling parameter Γ.

## 4. Summary

In this research, we investigated the effects of quantum screening on the Wigner time-delay for the polarization scattering in a dense semiclassical plasma. We employed the partial wave analysis to derive the phase shift for the polarization interaction in a partially ionized dense semiclassical plasma. In addition, we obtained the analytic expressions of the Wigner time-delay and the Wigner time-delay distribution for the electron–atom polarization interaction including the effects of quantum-mechanical character and plasma screening. We discovered that the quantum-mechanical character suppresses the Wigner time-delay as well as the Wigner distribution function for the polarization interaction in a dense semiclassical plasma. Moreover, we showed that the enhancement of plasma shielding strongly suppresses the Wigner time-delay and the Wigner distribution function. The quantum-mechanical character and the plasma screening would play significant roles in the study of the Wigner time-delay and the Wigner time-delay distribution. It has been shown that the wave packet dynamics is related to phase measurements of coherent optical signals [32]. In addition, it is also shown that the wave packet interferometry (WPI) [32,33] can detect the temporal envelopes of the pulses. In future experiments, it can be detected by the temporal development of the wave-packet related to the Wigner time-delay ${\bar{t}}_{\mathrm{Wigner}}$ since the Wigner time-delay is related to the gradient of the phase shift by using the wave packet interferometry. Moreover, Wigner time-delay distribution can also be detected since the Wigner time-delay distribution $P({\bar{t}}_{\mathrm{Wigner}})$ is related to the Wigner time-delay. Therefore, in the future, we may detect and resolve the temporal development of the wave-packet in a dense plasma using the wave packet interferometry. These results would provide useful information on the time-evolution and the energy dependence of the scattering system in a dense semiclassical plasma containing the quantum-mechanical character.
